# Supplementary material for: Identification of Epitopes on Rhinovirus 89 Capsid Proteins Capable of Inducing Neutralizing Antibodies
Source: Int J Mol Sci. 2022 May 4;23(9):5113. doi: 10.3390/ijms23095113 (PMC9100655; doi:10.3390/ijms23095113)
Supplement: Supplementary file 1 [file ijms-23-05113-s001.zip › ijms-1699022-supplementary.pdf]

Table S1. Recombinant VP1- and VP2-derived fragments and synthetic peptides of RV89.

Antigen	Amino Acid Sequence	Position	Length (aa)	MW (Da)
**89VP1-PI**	NPVENYIDSVLNEVLVVPNIQPSTSVSSHAAPALDAAETGHTSSVQPEDMIETRY<br>VITDQTRDETSIESFLGRSGCIAMIEFNTSSDKTEHDKIGKGFKT	1-100	100	10870
**89VP1-PII**	WKISLQEMAQIRRKYELFTYTRFDSEITIVTAAAAQGDDSGHIVLQFMYV<br>PPGAPVPEKRDDYTWQSGTNASVFWQEGQPYPRFTIPFMSIASAYYMFYD	101-200	100	11636
**89VP1-PIII**	GYDGDSAASKYGSVVTNDMGTICVRIVTSNQKHDLNIVCRIYHKAKHIKAW<br>CPRPPRAVAYQHTHSTNYIPSNGEATTQIKTRPDVFTGTNV	201-293	92	10174
**89VP1-p1**	NPVENYIDSVLNEVLVVPNIQPSTSVSSHAAPAC	1-33	34	3564
**89VP1-p2**	LDAAETGHTSSVQPEDMIETRYVITDQTRDETSC	34-66	34	3800
**89VP1-p3**	IESFLGRSGCIAMIEFNTSSDKTEHDKIGKGFKTWKISLQEMC	67-108	43	4867
**89VP1-p4**	WKISLQEMAQIRRKYELFTYTRFDSEITIVTAAC	101-133	34	4112
**89VP1-p5**	AAAQGDDSGHIVLQFMYVPPGAPVPEKRDDYTWQSGTNASVFC	133-174	43	4627
**89VP1-p6**	SGTNASVFWQEGQPYPRFTIPFMSIASAYYMFYDC	167-200	35	4076
**89VP1-p7**	ASAYYMFYDGYDGDSAASKYGSVVTNDMGTICVRIVTSNQKHC	192-233	43	4690
**89VP1-p8**	DLNIVCRIYHKAKHIKAWCPRPPRAVAYQHTHSC	234-266	34	4013
**89VP1-p9**	TNYIPSNGEATTQIKTRPDVFTGTNVC	267-292	27	2928
**89VP2-PI**	SPTVEACGYSDRLIQITRGDSTITSQDTANAVVAYGVWPSYLTPDDATAIDK<br>PTQPDTSSNRFYTLDSRSWTSASGWWWKLPDALKNMG	1-89	89	9805
**89VP2-PII**	IFGENMFYHFLGRSGYTIHVQCNSSKFHQGLLIVAAIPEHQLASATSGNVSV<br>GYNHTHPGEQGREVVPSRTSSDNKRPSDDSWLNFDGTL	90-179	90	9917
**89VP2-PIII**	LGNLPIYPHQYINLRTNNSATLILPYVNAVPMDSMLRHNNWSLVIIPICPLQ<br>VQPGGTQSIPITVSISPMFSEFSGPRSKVVFSTTQ	180-267	87	9623
**89VP2-p1**	SPTVEACGYSDRLIQITRGD	1-20	20	2181
**89VP2-p2**	ITRGDSTITSQDTANAVVAY	16-35	20	2083
**89VP2-p3**	AVVAYGVWPSYLTPDDATAI	31-50	20	2109
**89VP2-p4**	DATAIDKPTQPDTSSNRFYT	46-65	20	2228
**89VP2-p5**	NRFYTLDSRSWTSASSGWWW	61-80	20	2494
**89VP2-p6**	SGWWWKLPDALKNMGIFGEN	76-95	20	2350
**89VP2-p7**	IFGENMFYHFLGRSGYTIHV	91-110	20	2389
**89VP2-p8**	YTIHVQCNSSKFHQGLLIVA	106-125	20	2259
**89VP2-p9**	LLIVAAIPEHQLASATSGNV	121-140	20	2004
**89VP2-p10**	TSGNVSVGYNHTHPGEQGRE	136-155	20	2126
**89VP2-p11**	EQGREVVPSRTSSDNKRPSD	151-170	20	2244
**89VP2-p12**	KRPSDDSWLNFDGTLLGNLPI	166-186	21	2359
**89VP2-p13**	GNLPIYPHQYINLRTNNSAT	182-201	20	2287
**89VP2-p14**	NNSATLILPYVNAVPMDSMLR	197-217	21	2320
**89VP2-p15**	DSMLRHNNWSLVIIPICPLQV	213-233	21	2449
**89VP2-p16**	CPLQVQPGGTQSIPITVSIS	229-248	20	2025
**89VP2-p17**	TVSISPMFSEFSGPRSKVVFSTTQ	244-267	24	2620

Position: denotes the corresponding region of the native VP1/VP2 proteins. Length: indicates the number of amino acids. Underlined amino acids denote overlapping sequences between peptides.

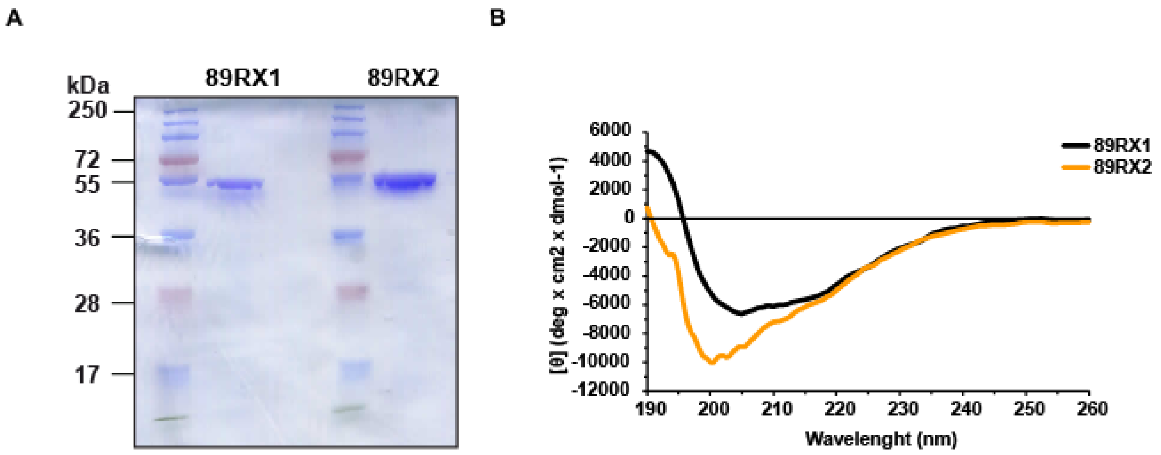

Figure S1. Purification and characterization of 89RX1 and 89RX2. (A) Coomassie-stained gel containing purified 89RX1 and 89RX2. Molecular weights (kDa) are indicated on the left margin. (B) Far-UV CD analysis of the purified PreS-based fusion proteins (i.e., 89RX1: black line; 89RX2: orange line). The spectra were recorded at 25°C and are expressed as mean residue ellipticities (Ø, y-axis) at given wavelengths (nm, x-axis).
